# The Relationship Between Harsh Parenting and Adolescents’ Pro-Environmental Behavior: A Moderated Mediation Model

**DOI:** 10.5334/pb.1344

**Published:** 2025-03-12

**Authors:** Dawei Wang, Shuangju Wei, Wenxu Mao, Ziying Lu, Yixin Hu

**Affiliations:** 1 School of Psychology, Shandong Normal University, CN

**Keywords:** harsh parenting, pro-environmental behavior, intrinsic motivation, egoistic values, altruistic values, biospheric values

## Abstract

**Background:**

Parents play a crucial role in cultivating adolescents’ pro-environmental behavior, which has attracted researchers’ attention. Nevertheless, the role of negative parenting styles has not been adequately concerned.

**Objectives:**

This research aimed to explore the influence of harsh parenting on adolescents’ pro-environmental behavior based on the Ecological Systems Theory, considering intrinsic motivation as a possible mediator and three types of values (egoistic, altruistic, and biospheric values) as possible moderators.

**Participants and Setting:**

Data were collected at two stages through self-report questionnaires filled in by 602 adolescents (40.2% boys) in China with an average age of 16.25 years.

**Methods:**

The questionnaires assessed pro-environmental behavior, harsh parenting, intrinsic motivation, and values. Research hypotheses were tested by Model 4 and Model 14 of the SPSS PROCESS macro.

**Results:**

Results showed that harsh parenting was negatively associated with adolescents’ pro-environmental behavior, which was mediated by intrinsic motivation. The interactions of three types of values and intrinsic motivation affected pro-environmental behavior differently. The relationship between intrinsic motivation and adolescents’ pro-environmental behavior was negatively moderated by egoistic values, but positively moderated by altruistic and biospheric values.

**Conclusions:**

The results revealed that harsh parenting was negatively and significantly correlated with adolescents’ pro-environmental behavior and such a relationship was mediated by intrinsic motivation and was moderated by values.

## Key points

**What is already known about this topic:**

1. Adolescents’ pro-environmental behavior might have a more lasting societal impact compared with that of adults.
2. Parenting styles can influence adolescents’ pro-environmental behavior.
3. Adolescence is a key period for the formation of motivation and values.

**What this topic adds:**

1. The negative effect of harsh parenting on adolescents’ pro-environmental behavior is discussed for the first time.
2. We uncovered the psychological process (i.e., adolescents’ intrinsic motivation) of harsh parenting affecting adolescents’ pro-environmental behavior.
3. The relationship between intrinsic motivation and pro-environmental behavior was moderated by egoistic values, altruistic and biospheric values.

## Introduction

Most current environmental problems are caused by human unfriendly behaviors to a certain extent, and the improvement of environmental quality must depend on people’s stable and lasting pro-environmental behavior (Karimi et al., 2021). Ever since the concept of pro-environmental behavior was proposed, adolescents’ pro-environmental behavior has been soon noticed by researchers because adolescents’ pro-environmental behavior might have a more lasting societal impact compared with that of adults (Poskus & Zukauskiene, 2017). Most studies have centered on the antecedents of adolescents’ pro-environmental behavior, including government policies (Yuan et al., 2020) and individual psychological factors (Lizin et al., 2017; López-Mosquera et al., 2015), while the role of parenting style is seriously underestimated. So far, only a few studies have explored the salutary effects of some positive parenting styles (e.g., autonomy-supporting, mindful, and authoritative parenting styles) on adolescents’ pro-environmental behavior (Grønhøj & Thøgersen, 2017; Guan & Geng, 2024; Ling & Chen, 2023), and the impairing effects of negative parenting styles on adolescents’ pro-environmental behavior are paid even less attention to. However, the family is the main place for children’s socialization (Wang & Fu, 2005), hence, negative parenting styles will hamper the socialization of adolescents’ attitudes and actions toward the environment to some degree.

As one of the typical negative parenting styles, harsh parenting has been demonstrated to be negatively correlated with adolescents’ prosocial behavior that is aimed at benefiting other individuals or groups (Streit et al., 2021). Similarly, pro-environmental behavior is targeted to benefit the environment, which ultimately will also benefit individuals reliant on the ecosystems (i.e., humanity). Klein et al. (2022) accordingly claimed that pro-environmental behavior can be regarded as one form of prosocial behavior. This opinion helps to disclose a possible relationship between harsh parenting and pro-environmental behavior. Further, drawing from the socialization theory, the negative self-cognition and aggressive behavior pattern stemming from harsh parenting may transmit to adolescents through the process of socialization (Degner & Dalege, 2013), which may be extended to the attitude and behavior toward the environment and finally reduce their tendency to take positive actions to protect the environment. Thus, it is well presumed that harsh parenting is negatively correlated with pro-environmental behavior.

According to the studies now available, the effect of parenting styles on adolescents’ behaviors (e.g., pro-environmental behavior) is often not direct but comes into play indirectly through certain psychological factors (Wang et al., 2018; Wang & Qi, 2017; Yue et al., 2021). Adolescents are at a sensitive stage of development, when adolescents’ autonomy and self-motivation are developing rapidly. Intrinsic motivation is a critical antecedent of adolescents’ pro-environmental behavior (Cerasoli et al., 2014; Grønhøj & Thøgersen, 2017), so it is crucial to develop adolescents’ intrinsic motivation. Grønhøj and Thøgersen (2017) have suggested that the autonomy-supporting parenting style, a kind of positive parenting style that provides gentle control and appropriate choice possibilities, plays a significant role in adolescents’ intrinsic motivation. On the contrary, harsh parenting may hinder the development of adolescents’ intrinsic motivation, then reducing adolescents’ pro-environmental behavior. Consequently, linking these research clues to examine how harsh parenting and intrinsic motivation affect adolescents’ pro-environmental behavior is necessary.

However, having a high intrinsic motivation does not necessarily mean actually engaging in pro-environmental behavior. Since values can serve as the guiding principles for selecting behaviors and, to some extent, are stable in varying situations (Schwartz, 1992), this study holds that values may be the key to bridging the gap between intrinsic motivation and the actual implementation of pro-environmental behavior. What’s more important, adolescence is also a key period for the formation of values (Eccles et al., 1989; Jodl et al., 2001), so to promote adolescents’ engagement in pro-environmental behavior, it is crucial to shape adolescents’ pro-environmental values during this period. Steg (2016) pointed out that values potentially impact intrinsic motivation, but it is unclear how these interactions between different values and intrinsic motivation would affect pro-environmental behavior.

The present study is concentrated on exploring the association between harsh parenting and adolescents’ pro-environmental behavior, meanwhile, examining whether this relationship could be mediated by intrinsic motivation and whether the effect could be moderated by values.

### Harsh parenting and adolescents’ pro-environmental behavior

Pro-environmental behavior is considered as the action performed to minimize adverse impacts on nature or improve environmental issues (Kollmuss & Agyeman, 2002; Steg & Vlek, 2009). In this study, pro-environmental behavior refers to people’s intention to protect the environment through various ways and the behavior that is beneficial to the environment in practice (Gong, 2008). The role of adolescents in environmental protection has become the focus of research in recent years (Grønhøj & Thøgersen, 2017; Krettenauer, 2017; Uitto et al., 2015), because the behavior habits of adolescents have strong plasticity and can be cultivated by the external environment (Eccles et al., 1989), and once formed, the behavior inclinations will remain stable throughout adulthood (Feather, 1995; Gong et al., 2022; Grønhøj & Thøgersen, 2009). Thus, the cultivation of and attention to adolescents’ pro-environmental behavior can bring about long-term and sustained benefits to the environment.

According to Ecological Systems Theory, parenting styles are generally believed as an important component in the family microsystem, and an important contextual variable that affects the development of adolescent behavior, and this influence is foundational and lasting (Bronfenbrenner, 1979). Similarly, adolescents’ stable and persistent pro-environmental behavior can be developed through the improvement of parenting styles. For example, Grønhøj and Thøgersen (2017) have found that adolescents’ self-determined motivation to engage in pro-environmental ways is rooted in the autonomy-supporting parenting style. However, a series of detrimental effects of negative parenting styles on adolescents’ pro-environmental behavior were paid less attention to.

Harsh parenting, one of the typical negative parenting styles, refers to a series of aggressive behaviors of parents toward children, mainly including physical aggression, verbal aggression, psychological aggression, and forced control behavior (Wang et al., 2016). According to the socialization theory, parents are crucial in adolescents’ socialization, guiding and helping them to integrate attitudes, values, beliefs and behaviors of the social culture into their actions (Degner & Dalege, 2013). However, harsh parenting increases adolescents’ negative cognition toward themselves, destroys their awareness of their suffering and decreases the wish and effort to relieve it (Liu & Hu, 2023); hence, in accordance with the socialization theory, this attitude of low concern for one’s own suffering induced by harsh parenting may be integrated and extended to the concept of the environment through the process of socialization, that is, adolescents may be indifferent to the suffering of and the threats confronted by the environment (Bengtsson et al., 2016), ultimately reducing adolescents’ intention to actively perform pro-environmental behavior. Besides, harsh parenting means adolescents are treated roughly, and this aggressive or damaging behavior pattern may also be passed on to adolescents through the process of socialization, making them use aggression as an approach to achieve personal goals and less likely to act in ways that protect the environment (Choi & Becher, 2019; Streit & Davis, 2022). Moreover, adolescents’ ability to internalize rules and cultivate conscience decreases (Kochanska, 1997), making it difficult for adolescents to form the moral norms and behavioral rules of environmental protection and engage in pro-environmental behavior. We speculate that harsh parenting can significantly negatively influence adolescents’ pro-environmental behavior. Consequently, we put forward the following hypothesis:

H1: Harsh parenting is negatively correlated with adolescents’ pro-environmental behavior.

### The mediating role of intrinsic motivation

Based on Self Determination Theory, intrinsic motivation is defined as “the innate tendency to engage in an activity for the sole pleasure and satisfaction derived from its practice” (Deci & Ryan, 1985). In this study, we focus on the intrinsic motivation toward the environment, which can be defined as individuals’ innate tendency to engage in pro-environmental behavior out of personal choice and interest, and for the sole pleasure and satisfaction derived from its practice (Pelletier et al., 1998). People driven by intrinsic motivation are more inclined to engage in activities out of their personal choice and interest and demonstrate greater persistence, wherein they can obtain satisfaction, intrinsic interest, and enjoyment (Deci & Ryan, 1985). When people are motivated intrinsically that environmental protection actions are critical to self-concept and collective interests, they are inclined to engage in pro-environmental behavior (Afsar et al., 2016). Previous studies have also demonstrated that an individual’s intrinsic motivation is positively correlated with pro-environmental behavior (Afsar et al., 2016; Grønhøj & Thøgersen, 2017; Pelletier et al., 1998; Renaud-Dubé et al., 2010). Accordingly, adolescents driven by intrinsic motivation are more active to participate in pro-environmental behavior.

According to the Self-Determination Theory, the maintenance and enhancement of intrinsic motivation needs supportive conditions, otherwise, it would be easily destroyed by non-supportive conditions (De Groot & Steg, 2010). Parenting styles as one of the important environmental conditions can affect the development of adolescents’ intrinsic motivation. The autonomy-promoting parenting style, as a supportive condition for providing independent choice, plays an important role in the cultivation of adolescents’ intrinsic motivation (Grønhøj & Thøgersen, 2017). Rather, when parents adopt harsh parenting, a destructive condition hindering autonomous development, they tend to respond to their children’s positive demands for autonomy with hostile control (Skowron et al., 2011) and lack effective satisfaction and guidance for their children’s emotional needs and behavior patterns, so adolescents’ perceptions of parental autonomy support are low (Lunkenheimer et al., 2017). In this case, adolescents are usually in a passive position rather than active initiators of some behaviors, which destroys adolescents’ behavioral autonomy, leading to a delay in self-directed development (Cicchetti & Lynch, 1993), and hindering the development of their intrinsic motivation.

In summary, the harsh parenting style reflects a high degree of parents’ control over adolescents, lacking autonomy support for adolescents, and more restrictive behaviors (Callahan et al., 2011), hindering the development of adolescents’ autonomy, which is not conducive to the cultivation of adolescents’ intrinsic motivation and then reduces the possibility of adolescents’ engagement in pro-environmental behavior. Therefore, we make the following hypotheses:

H2: Harsh parenting is negatively correlated with intrinsic motivation.

H3: Intrinsic motivation is positively correlated with pro-environmental behavior.

H4: Intrinsic motivation plays a mediating role between harsh parenting and adolescents’ pro-environmental behavior.

### The moderating roles of values

Schwartz (1992) defined values as: “a desirable transitional goal varying in importance, which serves as a guiding principle in the life of a person or other social entity”. Stern (2000) further believed that there are three different kinds of values: egoistic, altruistic, and biospheric values. Based on the Value-Basis Theory (Stern & Dietz, 1994), the perceived importance and evaluation of behavioral consequences rely on the values that people give priority to (Steg et al., 2014), and they are more likely to adhere to the goals consistent with their values (Sheldon & Elliot, 1999). Therefore, when people hold different values and give distinguished priorities to them, the above-mentioned transforming process of intrinsic motivation to pro-environmental behavior will be affected.

We respectively studied the moderating roles of three types of values. First, egoistic values signify that people rely on their costs and perceptions of benefits to decide whether to participate in pro-environmental behavior (De Groot & Steg, 2009), which is inconsistent with intrinsic motivation based on inner satisfaction and enjoyment. Adolescents with a high level of egoistic values put personal interests in the first place (De Groot & Steg, 2009), regardless of their enjoyment of behaviors, and thus pro-environmental behavior driven by intrinsic motivation will decrease. On the contrary, adolescents with a low level of egoistic values are less affected by behavioral benefits and costs and choose behaviors that conform to their intrinsic interest, which means that they are more inclined to participate in pro-environmental behavior driven by intrinsic motivation. In summary, egoistic values may weaken the influence of intrinsic motivation on adolescents’ pro-environmental behavior.

Second, altruistic values signify that people rely on the perceived costs and benefits for other people to decide whether to participate in pro-environmental behavior (Steg et al., 2014; Steg, 2016; De Groot & Steg, 2009). Therefore, adolescents with a high level of altruistic values are internally motivated to participate in pro-environmental behavior beneficial to others, from which they can obtain enjoyment and happiness (Steg, 2016). On the contrary, at a low level of altruistic values, adolescents are unlikely to feel happy and satisfied in altruistic behaviors (e.g. pro-environmental behavior), and they are unlikely to engage in pro-environmental behavior driven by intrinsic motivation. To sum up, altruistic values may strengthen the effect of intrinsic motivation on adolescents’ pro-environmental behavior.

Third, biospheric values signify that people rely on the perceived costs and benefits for the ecosystem and biosphere as a whole to decide whether to participate in pro-environmental behavior (De Groot & Steg, 2009). People with strong biospheric values will avoid environmental damage caused by their behaviors and they will be intrinsically motivated to participate in pro-environmental behavior (Steg, 2016; Stern et al., 1995). Therefore, adolescents with a high level of biospheric values attach more importance to the well-being of the biosphere and the environment (De Groot & Steg, 2009) and tend to participate in pro-environmental behavior based on intrinsic motivation. On the contrary, at a low level of biospheric values, adolescents pay less attention to environmental protection, and they are unlikely to take pro-environmental behavior as an approach to obtain the intrinsic satisfaction and pleasure, as well as to participate in pro-environmental behavior driven by intrinsic motivation. To sum up, biospheric values may also strengthen the effect of intrinsic motivation on adolescents’ pro-environmental behavior.

Based on the above inferences, three hypotheses were proposed:

H5a: Egoistic values negatively moderate the relationship between intrinsic motivation and adolescents’ pro-environmental behavior.

H5b: Altruistic values positively moderate the relationship between intrinsic motivation and adolescents’ pro-environmental behavior.

H5c: Biospheric values positively moderate the relationship between intrinsic motivation and adolescents’ pro-environmental behavior.

**Figure 1:**
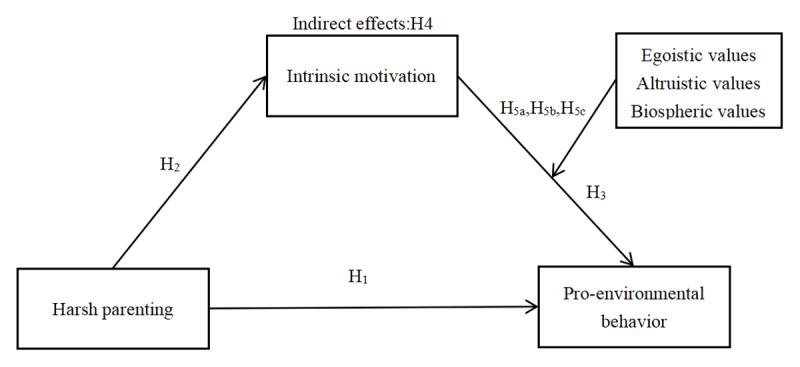
A moderated mediation model of the relationship between harsh parenting and adolescents’ pro-environmental behavior.

The structural model among variables in this research is shown in Figure 1.

## Methods

### Participants and procedure

Data were collected in two stages with a two-week interval to minimize the common method bias. Adolescents’ perceived harsh parenting and adolescents’ intrinsic motivation were measured in the first stage, and adolescents’ values and pro-environmental behavior were measured in the second stage. A total of 602 students completed questionnaires at both two stages. Their ages ranged from 15 to 19 years (*M* = 16.25, *SD* = 0.687) and the detailed characteristics of the sample are shown in Table 1. Power analysis based on linear multiple regression, eleven predictors, and a medium effect size (α = 0.05; *f*^2^ = 0.15), indicated 178 participants. Therefore, 602 participants are statistically adequate. All procedures adhere to the 1964 Helsinki Declaration and the ethical standards established by the research committee at the authors’ university. The participants provided written informed consent form voluntarily and anonymously and had the right to withdraw from the research program at any time.

**Table 1:** Characteristics of the sample (n = 602).


VARIABLES	CATEGORIES	FREQUENCY	PERCENTAGE (%)

Gender	Boy	242	40.2

	Girl	360	59.8

Grade	Senior one	387	64.3

	Senior two	215	35.7

Father’s education	Primary school and below	47	7.8

	Junior high school	231	38.4

	Technical secondary school/high school	176	29.2

	College/University and above	148	24.6

Mother’s education	Primary school and below	83	13.8

	Junior high school	243	40.4

	Technical secondary school/high school	162	26.9

	College/University and above	114	18.9

Monthly household income	<3000	67	11.1

	3001–5000	173	28.7

	5001–7000	166	27.6

	7001–9000	91	15.1

	>9001	105	17.4

Harsh parenting	Never (The mean is equal to 1)	91	15.1

	Mild (The mean is greater than 1 and less than or equal to 2)	326	54.1

	Moderate (The mean is greater than 2 and less than or equal to 4)	173	28.7

	Severe (The mean is greater than 4)	12	2.0

Intrinsic motivation	Low (The mean is less than or equal to 4)	29	4.8

	Moderate (The mean is greater than 4 and less than or equal to 6)	252	41.9

	High (The mean is greater than 6)	321	53.3


### Measures

Pro-Environmental Behavior. We used the 12-item scale developed by Gong (2008) and revised by Liu and Wu (2013) to measure pro-environmental behavior. A sample item was “Bring your shopping bag to grocery stores” on a 5-point Likert scale (1 = never, 5 = often) (Cronbach’s α = 0.894).

Harsh Parenting. We used the 4-item scale developed by Simons et al. (1991) and revised by Wang et al. (2020) to measure harsh parenting. A sample item was “when I did something wrong or made my parents angry, father (or mother) would lose temper or even yell at me” on a 5-point Likert scale (1 = never, 5 = always) (Cronbach’s α = 0.870).

Intrinsic Motivation. We used 4 items of Motivation Toward the Environment Scale (MTES) developed by Pelletier et al. (1998) to measure the intrinsic motivation of adolescents for environmental protection. A sample item was “Because I would feel pleasure in improving quality of environment” on a 7-point Likert scale (1 = completely disagree, 7 = completely agree) (Cronbach’s α = 0.898).

Values. We used the 12-item scale developed by De Groot and Steg (2008) and revised by Howell (2013) to measure values, including three dimensions: egoistic values(e.g., social power) (Cronbach’s α = 0.700), altruistic values(e.g., equality) (Cronbach’s α = 0.786), and biospheric values(e.g., preventing pollution) (Cronbach’s α = 0.876). Participants responded on a 5-point Likert scale (1 = strongly disagree, 5 = strongly agree). The result of the confirmatory factor analyses (CFA) indicated that the scale was well structured (*χ^2^/df* = 1.619, *RMSEA* = 0.032, *CFI* = 0.994, *TLI* = 0.989, *SRMR* = 0.022), with high validity and suitable for test in the Chinese adolescent population. All the used Likert scales in this study can be found in Appendix.

### Analysis

All measured core variables (i.e., harsh parenting, intrinsic motivation, egoistic, altruistic, and biospheric values, and pro-environmental behavior) are continuous variables. CFA was performed with Mplus8.3 software for verifying the discriminant validity of the six variables. Research hypotheses were tested by Model 4 and Model 14 of the SPSS PROCESS macro (Hayes, 2015). In addition, we found that there were correlations among gender, age, monthly household income, parents’ education, and main variables in structural model, so we statistically controlled for the above-mentioned variables in the following analysis.

## Results

### Measurement models

The results demonstrated that the six-factor model (*χ^2^/df* = 1.843, *RMSEA* = 0.037, *CFI* = 0.969, *TLI* = 0.961, *SRMR* = 0.061) fitted well. All Cronbach’s alpha coefficients ranged from 0.70 to 0.90, and the composite reliability (CR) values ranged from 0.80 to 0.95, which exceed the standard value of 0.60 (Bagozzi & Yi, 1988). The above results indicated that the measurement model had good reliability and validity for further analysis.

### Descriptive statistics and correlations

Table 2 summarizes all study variables’ descriptive statistics and bivariate correlations. As expected, harsh parenting was negatively correlated with intrinsic motivation and negatively correlated with adolescents’ pro-environmental behavior. Intrinsic motivation was positively correlated with adolescents’ pro-environmental behavior.

**Table 2:** Means, Standard Deviations and Correlations for the Variables.


	*M*	*SD*	1	2	3	4	5	6	7	8	9	10	11	12

1. Harsh parenting	1.923	.814	1											

2. Intrinsic motivation	6.104	.911	–.114**	1										

3. Egoistic values	3.462	.741	.090*	.078	1									

4. Altruistic values	4.429	.600	–.128**	.308**	.233**	1								

5. Biospheric values	4.552	.597	–.153**	.284**	.157**	.839**	1							

6. Pro-environmental behavior	3.800	.721	–.094*	.456**	.227**	.503**	.496**	1						

7. Age	16.250	.687	.045	–.198**	.019	–.055	–.058	–.071	1					

8. Grade			.074	–.110**	.037	–.135**	–.122**	–.085*	.670**	1				

9. Gender			–.043	.102*	–.098*	.079	.069	.010	–.019	.024	1			

10. Father’s education			–.016	.135**	.041	.090*	.108**	.190**	–.179**	–.246**	–.048	1		

11. Mother’s education			–.035	.112**	.048	.142**	.148**	.247**	–.179**	–.255**	–.058	.674**	1	

12. Household income			–.060	.066	–.009	.070	.098*	.061	–.078	–.099*	–.155**	.369**	.357**	1


Note: *N* = 602; *p < 0.05; **p < 0.01; ***p < 0.001; *SD* = Standard deviation.

### Mediating effect of intrinsic motivation

We tested if harsh parenting affected adolescents’ pro-environmental behavior via intrinsic motivation using SPSS PROCESS macro (model 4). After controlling for gender, age, grade, parents’ education, and monthly household income, harsh parenting can negatively and significantly predict adolescents’ pro-environmental behavior (β = –0.076, *SE* = 0.035, *p <* 0.05) (H1). Harsh parenting negatively and significantly affected intrinsic motivation (β = –0.114, *SE* = 0.045, *p <* 0.05) (H2). Intrinsic motivation positively and significantly affected adolescents’ pro-environmental behavior (β = 0.351, *SE* = 0.029, *p* < 0.001) (H3).

As shown in Table 3, harsh parenting affected adolescents’ pro-environmental behavior via intrinsic motivation. The mediating effect accounted for 51.948% of the total effect, and the confidence intervals did not include zero (H4).

**Table 3:** The total, direct and indirect pathways between harsh parenting and pro-environmental behavior.


EFFECT SOURCE	β	*SE*	95%CI	*T*	*P*	RELATIVE EFFECT VALUE

Total Effect	–0.076	0.035	[–0.146, –0.007]	–2.170	0.030	

Direct Effect	–0.036	0.032	[–0.099, 0.026]	–1.145	0.253	

HP→PEB						

Indirect Effect	–0.040	0.018	[–0.077, –0.007]			52.632%

HP→IM→PEB						


Note: HP = Harsh Parenting; IM = Intrinsic Motivation; PEB = Pro-Environmental Behavior.

### Moderating effect of values

Table 4 shows that the interaction term of intrinsic motivation and egoistic values negatively predicted pro-environmental behavior, but the interaction term of intrinsic motivation and altruistic values and the interaction term of intrinsic motivation and biospheric values positively predicted pro-environmental behavior separately.

**Table 4:** Test of the Moderating Effect of Values between Harsh Parenting and Pro-environmental Behavior.


VARIABLE	PRO-ENVIRONMENTAL BEHAVIOR											

	β	*SE*	*T*	95% CI	β	*SE*	*T*	95% CI	β	*SE*	*T*	95% CI

**Covariate**												

Age	0.072	0.050	1.463	[–0.025, 0.169]	0.021	0.046	0.455	[–0.070, 0.111]	0.034	0.046	0.740	[–0.057, 0.125]

Grade	–0.050	0.072	–0.700	[–0.191, 0.090]	0.051	0.067	0.759	[–0.081, 0.182]	0.030	0.067	0.455	[–0.101, 0.162]

Gender	–0.016	0.052	–0.316	[–0.118, 0.086]	–0.064	0.048	–1.324	[–0.158, 0.031]	–0.069	0.048	–1.433	[–0.164, 0.026]

Father’s education	0.004	0.036	0.107	[–0.068, 0.075]	0.028	0.034	0.823	[–0.039, 0.094]	0.019	0.034	0.565	[–0.047, 0.086]

Mother’s education	0.149	0.036	4.167***	[0.079, 0.219]	0.116	0.033	3.473***	[0.050, 0.181]	0.124	0.033	3.706***	[0.058, 0.189]

Family monthly income	–0.020	0.022	–0.931	[–0.063, 0.023]	–0.032	0.020	–1.570	[–0.071, 0.008]	–0.041	0.020	–2.015	[–0.081, –0.001]

**Independent Variable**												

HP	–0.059	0.031	–1.884	[–0.120, 0.003]	–0.008	0.029	–0.266	[–0.064, 0.049]	0.001	0.029	0.045	[–0.056, 0.057]

IM	0.342	0.029	12.005***	[0.286, 0.398]	0.280	0.028	10.045***	[0.225, 0.335]	0.284	0.028	10.243***	[0.229, 0.338]

**Moderator**												

EV	0.213	0.036	6.002***	[0.143, 0.282]								

AV					0.467	0.041	11.331***	[0.386, 0.548]				

BV									0.468	0.041	11.314***	[0.386, 0.549]

**Interaction**												

IM × EV	–0.127	0.042	–3.062***	[–0.209, –0.046]								

IM × AV					0.156	0.042	3.706***	[0.073,0.239]				

IM × BV									0.115	0.043	2.656**	[0.030, 0.200]

*R^2^*	0.299				0.398				0.392			

*F*	25.231***				39.107***				38.132***			


Note: HP = Harsh Parenting; IM = Intrinsic Motivation; EV = Egoistic Values; AV = Altruistic Values; BV = Biospheric Values.* *p* < 0.05; ** *p* < 0.01; *** *p* < 0.001.

Table 5 shows that the indirect effect was moderated by egoistic values. The moderated mediation index is 0.014. The 95% bootstrap confidence interval of difference in indirect effects did not include zero. We conducted a simple slope analysis, as Figure 2a shows, the influence of intrinsic motivation on pro-environmental behavior decreases as egoistic values increase, a negative interaction, meaning that lower levels of egoistic values led to a stronger association between intrinsic motivation and pro-environmental behavior (H5a).

**Table 5:** Test of conditional indirect effects.


EGOISTIC VALUES	PRO-ENVIRONMENTAL BEHAVIOR		

	β	*SE*	95%CI

Low-level EV (–1SD)	–0.050	0.024	[–0.100, –0.009]

High-level EV (+1SD)	–0.028	0.013	[–0.058, –0.005]

Indirect effect difference	0.022	0.014	[0.001, 0.056]


Note: Dependent Variable = Pro-environmental Behavior; EV = Egoistic Values.

**Figure 2a:**
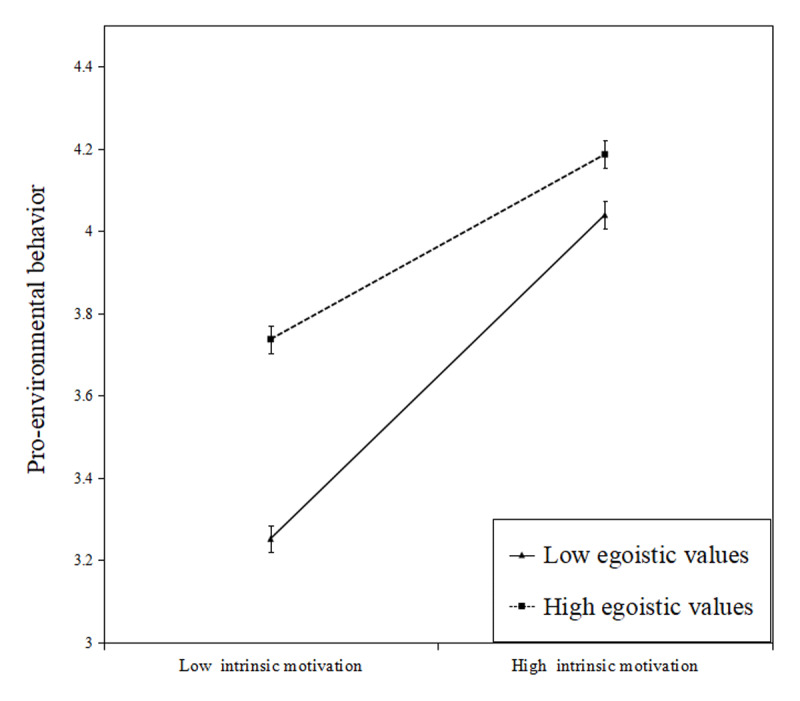
Moderating effect of egoistic values on the relationship between intrinsic motivation and pro-environmental behavior. (Error bars represent standard error.)

Table 6 shows that the indirect effect was moderated by altruistic values. The moderated mediation index is –0.018. The 95% bootstrap confidence interval of difference in indirect effects did not include zero. Figure 2b shows that the effect of intrinsic motivation on pro-environmental behavior increases as altruistic values increase, a positive interaction, meaning that higher levels of altruistic values led to a stronger association between intrinsic motivation and pro-environmental behavior (H5b).

**Table 6:** Test of conditional indirect effects.


ALTRUISTIC VALUES	PRO-ENVIRONMENTAL BEHAVIOR		

	β	*SE*	95%CI

Low-level AV (–1SD)	–0.021	0.011	[–0.046, –0.003]

High-level AV (+1SD)	–0.042	0.019	[–0.081, –0.007]

Indirect effect difference	–0.021	0.012	[–0.047, –0.002]


Note: Dependent Variable = Pro-environmental Behavior; AV = Altruistic Values.

**Figure 2b:**
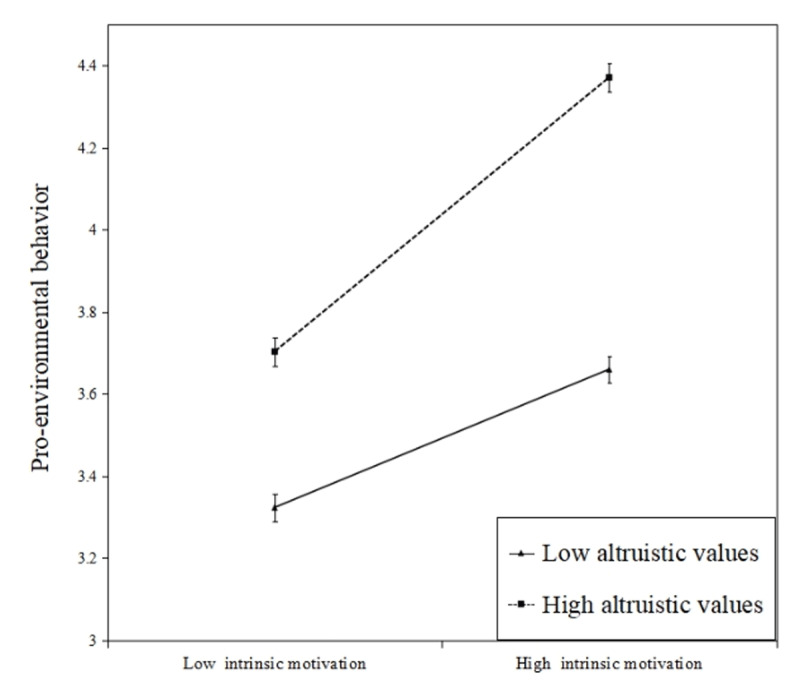
Moderating effect of altruistic values on the relationship between intrinsic motivation and pro-environmental behavior. Error bars represent standard error.

Table 7 shows that the indirect effect of intrinsic motivation was moderated by biospheric values. The moderated mediation index is –0.013. The 95% bootstrap confidence interval of difference in indirect effects did not include zero. Figure 2c shows that the effect of intrinsic motivation on pro-environmental behavior increases as biospheric values increase, a positive interaction, meaning that higher levels of biospheric values led to a stronger association between intrinsic motivation and pro-environmental behavior (H5c).

**Table 7:** Test of conditional indirect effects.


BIOSPHERIC VALUES	PRO-ENVIRONMENTAL BEHAVIOR		

	β	*SE*	95%CI

Low-level BV (–1SD)	–0.025	0.011	[–0.048, –0.005]

High-level BV (+1SD)	–0.038	0.018	[–0.076, –0.007]

Indirect effect difference	–0.014	0.010	[–0.037, –0.0003]


Note: Dependent Variable = Pro-environmental Behavior; BV = Biospheric Values.

**Figure 2c:**
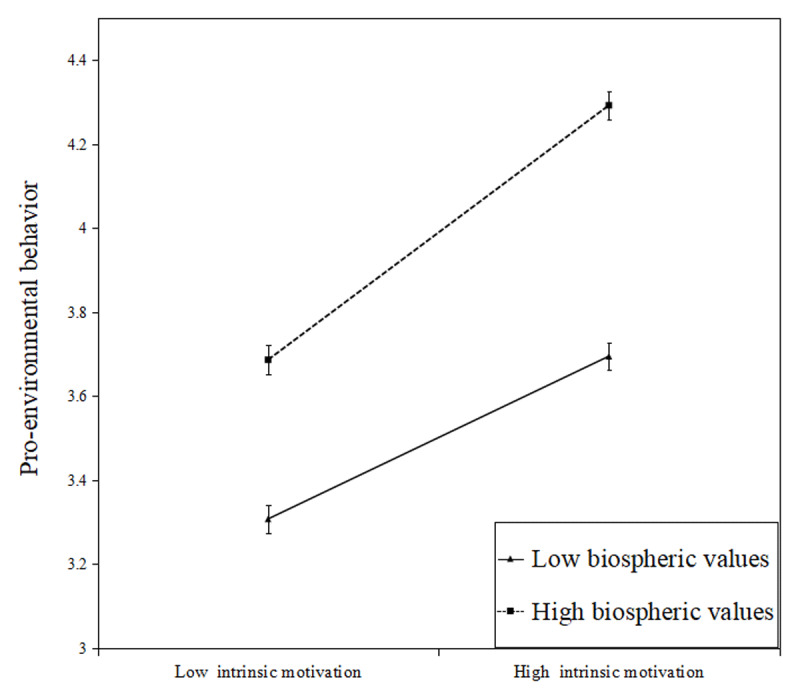
Moderating effect of biospheric values on the relationship between intrinsic motivation and pro-environmental behavior. Error bars represent standard error.

## Discussion

Increasing numbers of studies have demonstrated that family is an important place for the socialization of adolescents’ attitudes and actions on the environment and parents’ behavior can influence adolescents’ pro-environmental behavior (Gentina & Muratore, 2012; Gong et al., 2022). The association between parenting styles and adolescents’ pro-environmental behavior and its underlying mechanisms, however, have not been well studied. Inspired by previous researchers, the current study investigated the relationships between harsh parenting, intrinsic motivation, values, and adolescents’ pro-environmental behavior.

### Main findings

We found that harsh parenting can negatively affect adolescents’ pro-environmental behavior. This finding supports the ecological systems theory (Bronfenbrenner, 1979), that is, parenting styles in the family microsystem can affect adolescents’ behaviors. And more specifically, according to the socialization theory (Degner & Dalege, 2013), the influence of harsh parenting on adolescents’ pro-environmental behavior may occur through the socialization process, that is, harsh parenting causes adolescents to have a negative evaluation of themselves, lack of conscious awareness and concern to their painful experiences (Liu & Hu, 2023), and exposes them to aggressive behavior patterns (Wang et al., 2016). The aggressive behavior pattern and low concern for misfortune are internalized and generalized by adolescents to their attitudes and behaviors toward the environment in the process of socialization, thus reducing the implementation of pro-environmental behaviors. Besides, according to the results, the mean for harsh parenting is relatively low, which indicates that the sample of this study experienced a relatively low frequency or degree of harsh parenting but a certain percentage of teenagers still experience at least one kind of behavior belonging to the scope of harsh parenting. This is in line with Chinese context where harsh parenting is still one of the common styles of parenting, although the concept of family education is constantly updated with the development of social economy and culture (Ding et al., 2023). As the ancient Chinese proverb goes, “Spare the rod and spoil the child”. Additionally, the relatively high level of PEB among adolescents is closely linked to China’s promotion of ecological advancement and its focus on sustainable development. Influenced by such policy-driven initiatives, PEB is prevalent among Chinese adolescents. Overall, our findings highlight the importance of parents consciously controlling their potentially harsh parenting, as well as improving their parenting style and actively building positive interaction between themselves and their children, which helps increase adolescents’ pro-environmental behavior.

Regarding the mechanisms underlying the above-mentioned relationship, supporting our structural model, we found that harsh parenting affected adolescents’ pro-environmental behavior via intrinsic motivation. Our finding is in line with existing research that harsh parenting strongly inhibits the development of adolescents’ autonomy (Lunkenheimer et al., 2017), which is detrimental to the development of intrinsic motivation for pro-environmental behavior and hinders adolescents’ engagement in pro-environmental behavior. Moreover, according to Self-Determination Theory (Deci & Ryan, 1985), environment that supports autonomy will facilitate more strong intrinsic motivation, which will promote adolescents’ sustained engagement in pro-environmental behavior (Grønhøj & Thøgersen, 2017; Pelletier et al., 1998; Renaud-Dubé et al., 2010). Thus, the higher the level of harsh parenting, the less likely adolescents were to engage in pro-environmental behavior based on intrinsic motivation. In addition, we separately explored the moderating roles of the three types of values (egoistic, altruistic, and biospheric values).

First, our findings indicate that egoistic values negatively moderated the relationship between intrinsic motivation and adolescents’ pro-environmental behavior. Under the influence of egoistic values, individuals tend to engage in pro-environmental behavior out of the desire to demonstrate personal morality rather than driven by pure intrinsic motivation (Steg et al., 2014), thus the promoting effect of intrinsic motivation on pro-environmental behavior will be weakened. In contrast, at the low level of egoistic values, individuals engage in pro-environmental behavior with less consideration of personal costs, less tendency to obtain self-interests, and more attention to the environment. At this point, the promoting effect of intrinsic motivation on pro-environmental behavior will be enhanced, and adolescents are intrinsically motivated and self-satisfied due to the environmental gains they bring about (De Groot & Steg, 2009). Besides, the effect of egoistic values on pro-environmental behavior is positive, which is in line with the statement in existing research that, in some cases, stronger egoistic values may also encourage people to support and take pro-environmental action (Bouman et al., 2021). However, excessive egoistic values are still not worth advocating, because individuals with egoistic values perform pro-environmental behavior for the purpose of pursuing personal status or interests, which is not conducive to the continuous promotion of pro-environmental behavior.

Second, we found that the relationship between intrinsic motivation and adolescents’ pro-environmental behavior was positively moderated by altruistic values, which is probably because when adolescents have a high level of altruistic values, they tend to engage in pro-environmental behavior for others’ interests, obtaining more pleasure and psychological satisfaction (De Groot & Steg, 2009), which promotes individuals to engage in more pro-environmental behavior based on intrinsic motivation.

Third, biospheric values also positively moderated the relationship between intrinsic motivation and adolescents’ pro-environmental behavior. This is because engaging in pro-environmental behavior conforms to the behavioral goals of biospheric values (De Groot & Steg, 2008; 2009). Adolescents with a high level of biospheric values become more aware of their behaviors that may cause environmental problems (Steg, 2016; Stern et al., 1995), and are more inclined to derive pleasure from engaging in pro-environmental behavior, promoting pro-environmental behavior based on intrinsic motivation.

What’s more, the results showed that egoistic, altruistic and biosphere values moderated the indirect path from harsh parenting to pro-environmental behavior via intrinsic motivation. The higher altruistic and biosphere values and the lower egoistic values, the stronger the mediating role of intrinsic motivation. It indicates that higher altruistic and biosphere values will magnify the harmful effect of the reduced intrinsic motivation induced by harsh parenting on pro-environmental behavior, while higher egoistic values will weaken such effect. For individuals with high altruistic or biosphere values, they care about the interests of others or the biosphere as a whole (De Groot & Steg, 2009), that is, they perform pro-environmental behavior for the sake of benefiting others (such as family, descendants) and the whole ecological environment. However, under the harsh parenting context, without enough support of intrinsic motivation, such altruistic or biosphere principles of individuals are difficult to be translated into actual pro-environmental behavior. More specifically, if the adolescents are exposed to harsh parenting, they may form a negative self-evaluation (Liu & Hu, 2023) and lack of autonomy (Lunkenheimer et al., 2017), causing a low level of intrinsic motivation to environmental protection and hence being not able to obtain pleasure and satisfaction from those behaviors. Hence, the adolescents who experienced harsh parenting but have altruistic or biosphere values may fall into a cognitive dilemma, that is, they think they have a positive moral image since school or social education has cultivated them to have altruistic or biosphere values, but they can’t even derive inner pleasure and satisfaction from pro-environmental behavior, which further makes the adolescents doubt their positive self-cognition and lose confidence, and leads to more doubt and even withdrawal before individuals actually perform pro-environmental behavior. For individuals with high egoistic values, they are concerned about their own interests (De Groot & Steg, 2009). For example, they may think that environmental pollution can cause harm to their own health, so in this case, adolescents with egoistic values will still perform some pro-environmental behavior to protect the interests of themselves, regardless of the reduction of intrinsic motivation induced by harsh parenting.

### Limitations and future research

The following aspects of this study remain limited. First, we did not distinguish between public and private pro-environmental behavior in the study. Different types of pro-environmental behavior may lead to different research results. Future research can further categorize the different types of pro-environmental behavior to verify the research results.

Second, our results are based on self-reported data. Although the data were obtained at two-time points to minimize the common method bias, we cannot rule out the influence of social desirability and other subjective deviations on the responses owing to the nature of self-reporting tests. Therefore, future studies could collect data using multi-agent (e.g., parental reports) evaluations.

Third, the current research cannot infer the causal relationship, in the future, researchers can consider using experimental research or longitudinal research to make causal conclusions. Additionally, participants in this study were limited to adolescents from China, which may restrict the generalizability of the findings. Future studies could expand the sample to include individuals from diverse cultural backgrounds, thereby enhancing the generalizability of the conclusions.

Fourth, other potential mechanisms between harsh parenting and adolescents’ pro-environmental behavior and other parenting styles that may affect pro-environmental behavior can be further investigated. Moreover, because of the differences in personality traits (e.g., responsibility, environmental locus of control) of adolescents, their perceptions of parents’ attitudes and behaviors are also varied, which should be considered in future research. Additionally, recent studies have found that neighbourhood peers’ pro-environmental behaviors can positively affect that of focal adolescents (Wang et al., 2021), while high hedonistic values may hamper adolescents’ environmental behaviors (Bouman et al., 2021; Steg et al., 2014); hence, future relevant studies can also take the peer influence, more relevant value orientations and other potential factors into account.

### Implications

We are first to focus on the relationship between harsh parenting and adolescents’ pro-environmental behavior based on the Ecological Systems Theory and Socialization Theory, which expands the study field of adolescents’ pro-environmental behavior. Furthermore, it is worth mentioning that harsh parenting may have intergenerational transmission effects (Simons et al., 1991). Adolescents regard their parents as learning objects and those who have experienced harsh parenting may have less concern for others and the whole environment and use aggression as a means to achieve personal goals (Choi & Becher, 2019; Streit & Davis, 2022), which destroys the cultivation of pro-environmental behavior in the next generation. It is hard to imagine that parents engaging in harsh parenting might hinder their children to behave in ways that are good for the environment, and our results provide a new theoretical perspective for such a relationship.

In addition, this study indicates that the external environment is important for the development of intrinsic motivation of adolescents, and our research highlights the importance of parenting styles in cultivating intrinsic motivation, thus promoting pro-environmental behavior. Moreover, values and intrinsic motivation are discussed in empirical studies in the domain of pro-environmental behavior, but they are usually explored separately (Howell, 2013; De Groot & Steg, 2010). We study the moderating roles of three types of values, which enriches the empirical research on the impact of intrinsic motivation and values on pro-environmental behavior. The findings provide direction for future intervention strategies by showing how three different types of values interact with intrinsic motivation in different ways to influence pro-environmental behavior. Our findings also have practical implications. Environmental protection policymakers should attach importance to family education in environmental protection when addressing global ecological and environmental issues. Except for implementing green development policies, for adolescents, parents should adopt positive parenting styles and actively promote family education on ecological civilization. Moreover, we should emphasize the development of adolescents’ intrinsic motivation for pro-environmental behavior. As a typical autonomous motivation (Gagné & Deci, 2005), intrinsic motivation makes individuals show stronger persistence in performing tasks (Cerasoli et al., 2014). As for parents, they should avoid the occurrence of harsh parenting, and give more autonomy and emotional support to adolescents for cultivating their intrinsic motivation.

In addition, these findings suggested that altruistic and biospheric values play important roles in adolescents’ engaging in pro-environmental behavior. Since adolescents are forming value systems (Steg, 2016), we should encourage them to act based on altruistic and biospheric values and emphasize the cultivation of these two values (De Groot & Steg, 2009), which can be done by increasing the cognitive accessibility and the relative importance of these two values (De Groot & Steg, 2009).

## Conclusion

In summary, we found that harsh parenting can negatively and significantly affect adolescents’ pro-environmental behavior and such a relationship was mediated by intrinsic motivation. We combined intrinsic motivation with three types of values to examine how they jointly affected pro-environmental behavior. The relationship between intrinsic motivation and adolescents’ pro-environmental behavior was negatively moderated by egoistic values, while the relationship was positively moderated by altruistic and biospheric values.

These findings help to better understand the relationship and underpinning mechanisms between harsh parenting and adolescents’ pro-environmental behavior, which is of great significance for advancing adolescents’ pro-environmental behavior and improving environmental quality.

## Data Accessibility Statement

The datasets generated during and/or analysed during the current study are available from the corresponding author on reasonable request.

## Additional File

The additional file for this article can be found as follows:

10.5334/pb.1344.s1 Appendix. Measurement Tools Used in the Study.
