# Supplementary material for: The Relationship Between Harsh Parenting and Adolescents’ Pro-Environmental Behavior: A Moderated Mediation Model
Source: Psychol Belg. 2025 Mar 12;65(1):54–68. doi: 10.5334/pb.1344 (PMC11908419; doi:10.5334/pb.1344)
Supplement: Appendix. — Measurement Tools Used in the Study. [file pb-65-1-1344-s1.pdf]

Appendix

**Harsh parenting.** Please rate the extent to which you agree with the following descriptions according to your actual situation with your parents, and choose the corresponding number.		Never	Hardly ever	Sometimes	Often	Always
1	When I did something wrong or made my parents angry, father (or mother) would lose temper or even yell at me.	1	2	3	4	5
2	When I did something wrong or made my parents angry, father (or mother) would hit me with something.	1	2	3	4	5
3	When I did something wrong or made my parents angry, father (or mother) would hit me with his/her hand or kick me with his/her foot.	1	2	3	4	5
4	When I did something wrong or made my parents angry, father (or mother) would tell me to get out or lock me out of the house.	1	2	3	4	5

**The intrinsic motivation toward the environment.** Why are you doing things for the environment? Here are some possible reasons why you might be doing something for the environment. Please choose the answer to each question that matches your actual idea.		Completely disagree	Relatively disagree	Somewhat disagree	Uncertain	Somewhat agree	Relatively agree	Completely agree
1	Because I would feel pleasure in mastering new ways to help.	1	2	3	4	5	6	7
2	Because I would feel pleasure in improving quality of environment.	1	2	3	4	5	6	7
3	Because I like feeling when doing things for environment.	1	2	3	4	5	6	7
4	Because I would feel pleasure in contributing to environment.	1	2	3	4	5	6	7

**Values.** There is no right or wrong answer for the following questions. Please rate the importance of each value	Strongly disagree	Disagree	Not for sure	Agree	Strongly agree

“as a guiding principle in your life” according to your actual situation and choose the corresponding option.						
1	Social power (control over others, dominance).	1	2	3	4	5
2	Wealth (material possessions, money).	1	2	3	4	5
3	Authority (the right to lead or command).	1	2	3	4	5
4	Influential (having an impact on people and events).	1	2	3	4	5
5	Equality (equal opportunity for all).	1	2	3	4	5
6	A world at peace (free of war and conflict).	1	2	3	4	5
7	Social justice (correcting injustice, care for the weak).	1	2	3	4	5
8	Helpful (working for the welfare of others).	1	2	3	4	5
9	Preventing pollution (protecting natural resources).	1	2	3	4	5
10	Respecting the earth (harmony with other species).	1	2	3	4	5
11	Unity with nature (fitting into nature).	1	2	3	4	5
12	Protecting the environment (preserving nature).	1	2	3	4	5

1

**Pro-environmental behavior.** Have you had any of the following behaviors in the past year? Please read the description below carefully and choose the answer for each question according to your actual situation.		Nev er	Seld om	Occasio nally	Some times	Ofte n
1	Publicly express (e.g., speech, writing, etc.) support for environmental protection.	1	2	3	4	5
2	Discuss environmental protection issues with friends and relatives.	1	2	3	4	5
3	Save and reuse plastic shopping bags.	1	2	3	4	5
4	Actively participate in environmental protection activities sponsored by schools or environmental protection organizations.	1	2	3	4	5
5	Bring your shopping bag to grocery stores.	1	2	3	4	5
6	Actively participate in various forms of environmental protection publicity and education activities.	1	2	3	4	5

7	Actively pay attention to environmental protection and information in the media.	1	2	3	4	5
8	When the room is empty, leave the room to turn off the lights or fans (air conditioner).	1	2	3	4	5
9	Accumulate empty beverage bottles, wine bottles, etc., and sell them.	1	2	3	4	5
10	Advise others to stop damaging the environment (e.g., littering, discharging sewage, etc.).	1	2	3	4	5
11	Reuse the other side of waste paper and printing paper.	1	2	3	4	5
12	Try not to use disposable personal items.	1	2	3	4	5
